# Facility readiness in low and middle-income countries to address care of high risk/ small and sick newborns

**DOI:** 10.1186/s40748-019-0105-9

**Published:** 2019-06-18

**Authors:** Indira Narayanan, Jesca Nsungwa-Sabiti, Setyadewi Lusyati, Rinawati Rohsiswatmo, Niranjan Thomas, Chinnathambi N. Kamalarathnam, Jane Judith Wembabazi, Victoria Nakibuuka Kirabira, Peter Waiswa, Santorino Data, Darious Kajjo, Paul Mubiri, Emmanuel Ochola, Pradita Shrestha, Ha Young Choi, Jayashree Ramasethu

**Affiliations:** 1 Georgetown University Medical Center, Washington, DC, USA; 2 Ministry of Health, Kampala, Uganda; 3 Harapan Kita Women and Children’s Hospital, West Jakarta, Indonesia; 4 Cipto Mangunkusumo Hospital, Universitas Indonesia, Jakarta, Indonesia; 5 Christian Medical College, Vellore, Tamil Nadu India; 6 Institute of Child Health, Chennai, Tamil Nadu India; 7 Nsambya Hospital, Kampala, Uganda; 8 Department of Health Policy, Planning and Management, Makerere University School of Public Health, Kampala, Uganda; 9 Global Health Division, Karolinska Institutet, Stockholm, Sweden; 10 Leader Makerere University Maternal and Newborn Centre of Excellence, Kampala, Uganda; 11 Department of Pediatrics and Child Health, Mbarara University of Science and Technology, Mbarara, Uganda; 12 Makerere University School of Public Health, Kampala, Uganda; 13 Department of HIV, Research and Documentation, St. Mary’s Hospital Lacor, Gulu, Uganda; 14 MedStar Georgetown University Hospital, Washington, DC, USA

**Keywords:** Newborn, Preterm babies, Prematurity, Low birthweight, Facility-based care, Special care newborn units, Level II newborn care, Equipment for newborn care, Commodities for newborn care, Low and middle-income countries, Health policies

## Abstract

**Background:**

The successful promotion of facility births in low and middle-income countries has not always resulted in improved neonatal outcome. We evaluated key signal functions pertinent to Level II neonatal care to determine facility readiness to care for high risk/ small and sick newborns.

**Method:**

Facility readiness for care of high risk/ small and sick babies was determined through self-evaluation using a pre-designed checklist to determine key signal functions pertinent to Level II neonatal care in selected referral hospitals in Uganda (10), Indonesia (4) and India (2) with focus on the Sub-Saharan country with greater challenges.

**Results:**

Most facilities reported having continuous water supply, resources for hand hygiene and waste disposal. Delivery rooms had newborn corners for basic neonatal resuscitation, but few practiced proper reprocessing of resuscitation equipment. Birth weight records were not consistently maintained in the Ugandan hospitals. In facilities with records of birth weights, more than half (51.7%) of newborns admitted to the neonatal units weighed 2500 g or more. Neonatal mortality rates ranged from 1.5 to 22.5%. Evaluation of stillbirths and numbers of babies discharged against medical advice gave a more comprehensive idea of outcome. Kangaroo Mother Care was practiced to varying extents. Incubators were more common in Africa while radiant warmers were preferred in Indian hospitals. Tube feeding was practiced in all and cup feeding in most, with use of human milk at all sites. There were proportionately more certified pediatricians and nurses in Indonesia and India. There was considerable shortage of nursing staff, (worst nurse –bed ratio ranging from 1 to 15 in the day shift, and 1 to 30 at night). There was significant variability in facility readiness, as in data maintenance, availability of commodities such as linen, air -oxygen blenders and infusion pumps and of infection prevention practices.

**Conclusions:**

Referral neonatal units in LMIC have challenges in meeting even the basic level II requirements, with significant variability in equipment, staffing and selected care practices. Facility readiness has to improve in concert with increased facility births of high risk newborns in order to have an impact on neonatal outcome, and on achieving Sustainable Development Goals 3.2.2.

**Electronic supplementary material:**

The online version of this article (10.1186/s40748-019-0105-9) contains supplementary material, which is available to authorized users.

## Introduction

Over the last two decades, births of newborn infants in health care facilities in low and middle-income countries (LMICs) have increased progressively, from 60% in 1990 to 75% between 2010 and 2015 [1]. However, this increase has not always been associated with improved outcomes [2, 3]. Tools for facility-based care have mainly addressed basic essential newborn care such as hand hygiene, other clean delivery practices, temperature maintenance, clean cord care, early and exclusive breastfeeding, identification of danger signs with appropriate care seeking, basic resuscitation, Kangaroo Mother Care (KMC) and early postnatal care [4, 5]. However, more comprehensive facility-based care of what are termed as ‘small and sick babies’ is addressed to a lesser extent, although recent revised recommendations for maternal and newborn care at the global level have begun to consider additional components [6].

In the authors’ experiences, in facilities for “small and sick babies” in LMIC, the care, resources and outcomes can be highly variable, and challenges considerable. This article focuses on some of the key issues of extra or ‘special’ care for these newborns in what are called “Special Care Neonatal Units” or equivalent to level II care, in some countries [7, 8]. This constitutes the basic requirement for the care of small and sick babies and is essential in all hospitals in LMICS, at the level of district hospitals and above.

## Methods

In this preliminary evaluation, neonatal units in referral hospitals in Uganda, Indonesia and India were studied for readiness to provide care for the newborn. The countries served as examples of LMIC countries with more focus on a Sub-Saharan African country (Uganda), where challenges are often greater, and additionally in South Asia (India) and South East Asia (Indonesia). The hospitals included government (public) and private not-for-profit facilities. These hospitals are considered to be referral hospitals, which accept small or sick babies born at home or in sub-district/peripheral health centers. A checklist-style excel table (Additional file 1: Table S1) was adapted from the American Academy of Pediatrics recommendations (2012) and the National Neonatology Forum of India by the authors (IN and JR) with emphasis on signal functions essential for care of small and sick babies [8, 9]. Key signal functions included provision of extra warmth for babies who did not receive continuous Kangaroo Mother Care (KMC) through incubators or radiant warmers, intravenous fluids, alternate methods of feeding including tube feeding, safe oxygen use, and respiratory support up to continuous positive airway pressure (CPAP). Other elements included types and numbers of health care workers, proportion related to bed strength, selected components of infection prevention, and availability of antibiotics. Items such as ventilators were not covered as the focus was on level II or intermediary care that could address the basic signal functions for the “small and sick newborns***”.*** The tool was pilot tested and appropriate changes incorporated. Information was self-reported by the authors for the period Jan 1 to Dec 31, 2017. Necessary clearances were obtained for collection of data and information. Data related to neonatal deaths and stillbirths were obtained from the facilty registers and from forms in which data was collected for district and national health information systems.

Sixteen hospitals were included: 10 facilities in Uganda (7 government/public and 3 private-not-for-profit, 1 tertiary, 2 regional and 7 district hospitals), 4 public facilities in Indonesia (2 tertiary, 1 regional and 1 district), and 2 tertiary regional hospitals in India (1 private not for profit and 1 public hospital). There was an ongoing study of district hospitals in India when this study was planned, and therefore we could not include district hospitals. We still included these two tertiary hospitals to document whether they had at least the components of level II/special care for the high risk/small and sick babies.

In all the hospitals, the high risk/ small and sick babies were cared for in what were locally termed as neonatal intensive care units. They were found to be so variable that in this study they are termed as “neonatal units” (NU). Information documented, however, related only to the signal functions and issues relevant to level II newborn care or special care neonatal units (SCNU).

## Results

### Patient load and outcome

Table 1 presents the number of deliveries and admissions to the NUs along with outcomes. The annual numbers of live births ranged from 951 to 14,469. All hospitals had in- house deliveries, except one hospital in India (NU#16), that was a large children’s hospital with only outborn admissions. All the hospitals except one (#2) reported having ambulances for transporting babies to other facilities where required, but were not used to pick up babies.

**Table 1:** Hospital Deliveries, Neonatal Unit Admissions and Outcomes

Type of Hospital	1.UG- ^d^ Tertiary Urban	2.UG- Public Regional Urban	3.UG- Public Regional Urban	4.UG- Public District Urban	5.UG- Public District Urban	6.UG- Public District Rural	7.UG- Public District Rural	8.UG- Public District Rural	9.UG- ^d^ District Rural	10.UG- ^d^ District Rural	11. INDO- Public Tertiary Urban	12. INDO- Public Tertiary Urban	13. INDO- Public RegionalUrban	14. INDO- Public District Urban	15. INDIA- ^d^ Tertiary Urban	16. INDIA- Public Tertiary Urban
Total Births	5105	8569	5689	6506	5605	3206	2768	2276	2122	1002	1719	1644	3209	3109	14,710	X
Live Births	5013	8247	5468	6224	5486	3109	2659	2183	1938	951	1608	1524	3179	2989	14,469	X
Total Still Births (no/1000 total births)	92 (18 per 1000)	322 (38 per 1000)	221 (39 per 1000)	282 (43 per 1000)	119 (21 per 1000)	97 (30 per 1000)	109 (39 per 1000)	93 (41 per 1000)	184 (87 per 1000)	51 (51 per 1000)	111 (65 per 1000)	120 (73 per 1000)	30 (9 per 1000)	120 (39 per 1000)	241 (16 per 1000)	X
Fresh SB (% of total SB)	38 (41.3)	164 (50.9)	114 (51.6)	137 (48.6)	78 (65.5)	47 (48.5)	61 (56.0)	45 (48.4)	111 (60.3)	31 (60.8)	NA	NA	NA	NA	NA	X
Annual Admissions to NU	2463	1729	1707	1164	506	1586	240	300	180	156	1600	1028	970	1900	3115	2188
Inborn Babies (% of live births)	2178 (43.4)	NA	1603 (29.3)	NA	279 (5.1)	1586 (51.0)	NA	NA	180 (9.3)	156 (16.4)	1269 (78.9)	893 (58.6)	691 (21.7)	1311 (43.9)	2798 (19.3)	X
Out-born Babies (% of NU admissions)	285 (11.6)	NA	104 (6.1)	NA	227^b^ (44.9)	^c^	NA	NA	^c^	^c^	429 (26.8)	135 (13.1)	279 (28.8)	589 (31.0)	317 (10.2)	2188 (100)
Babies < 2500 g (% of admissions)	NA^a^	422 (24.4)	705 (41.3)	NA^a^	NA^a^	NA	NA	NA	NA	NA	824 (51.5)	758 (73.7)	373 (38.5)	999 (52.6)	1746 (56.1)	1054 (48.2)
Neonatal Deaths (% of admissions)	115 (4.7)	106 (6.1)	94 (5.5)	156 (13.4)	114 (22.5)	45 (2.8)	48 (20.0)	24 (8.0)	34 (18.9)	24 (15.4)	70 (4.4)	82 (8.0)	91 (9.4)	242 (12.7)	46 (1.5)	383 (17.5)
“Discharged against medical advice” (% of admissions)	NA	NA	341 (20)	NA	10 (2)	NA	NA	NA	NA	NA	8 (0.5)	NA	4 (0.4)	NA	89 (2.9)	62 (2.8)

Hospitals # 2, 4, 7 and 8 maintained data only on the *total* number of admissions in the neonatal unit and not on the numbers of inborn and out-born babies

NA - Not available; X – Not applicable; ^a^ - Information relevant to birth weight was not available for all babies from the hospital record sections, hence percentage of babies below 2500 g. was not calculated for these centers; ^b^- out-born newborns with suspected infection, sent to pediatric ward; ^c^- All out-born admissions are sent to pediatric ward; ^**d**^ - Private, Not for Profit Hospital; *UG* Uganda, *Indo* Indonesia

The total stillbirth (SB) rate ranged from 18 to 87/1000 total births (mean = 41/1000 total births) in the Ugandan hospitals, 9 to 73/1000 total births (mean = 47/1000 total births) in the Indonesian hospitals, and 16/1000 total births in the Indian hospital with deliveries. While the total numbers of SBs were available in all the hospitals where deliveries took place, data on intrapartum or fresh stillbirths were available only for the facilities in Uganda (range: 41.3–65.5.% of the total SBs; mean 53.2%) .

All hospitals maintained data on numbers of inborn and out-born babies except for 4 in Uganda (hospitals # 2, 4, 7 and 8 who just had data on the total number of admissions). A significant proportion of inborn babies were admitted to the NU, ranging from 5.1% to as high as 78.9% (#11 in Indonesia) of all live births. In hospitals with a high proportion of inborn admissions, the admissions also included infants admitted to “allow the mothers to rest”. Data about birth weights of babies admitted to NU was consistently available only for 2 facilities (# 2 and #3) in Uganda. The proportion of babies admitted to the NU with a birth weight below 2500 g ranged from 24.4 to 73.7% (mean 48.3%).

In most of the hospitals studied, outborn babies were admitted to the NUs, but in 5 district hospitals in Uganda, such babies, especially those who were obviously sick, were sent to the pediatric ward and cared for either in separate cubicles or along with the older infants and children. Although data related to these babies were maintained with the other children, even at times disaggregated by age, the staff, when questioned, felt that there were potential risks of information on these out-born babies in the Pediatric ward not getting incorporated into data related to the newborn in the health information system.

The mortality rates of neonates admitted to the NU ranged from 1.5 to 22.5%, (Uganda- 2.8 to 22.5% and in Indonesia 4.4 to 12.7%.). In the two Indian facilities, the rates were 1.5% in # 15 that admitted both inborn and out-born babies and 17.5% in hospital # 16 that admitted only out-born babies.

Table 1 also shows data from 6 hospitals on the number of babies who were discharged against medical advice (DAMA). Two hospitals in Uganda reported rates of 20 and 2% of all admissions, two hospitals in Indonesia reported DAMA rates of 0.5 and 0.4%, and the two hospitals in India reported rates of 2.9 and 2.8%. Data regarding babies referred to other hospitals were not available, except in hospital # 13 in Indonesia, that referred 50 babies to another facility.

Table 2 shows staffing patterns in the NU, indexed to the bed strength. Staffing varied widely, with nurse –bed ratio ranging from 0.7 to 15 beds in the day shift, and 1 to 30 in the night shift. The hospitals with the least number of beds per nurse of 0.7 and 1 were in district hospitals in Uganda that catered to far fewer beds/babies.

**Table 2:** Neonatal Unit Beds and Staff

Type of Hospital	1.UG- ^a^ Tertiary Urban	2.UG- Public Regional Urban	3.UG- Public Regional Urban	4.UG- Public District Urban	5.UG- Public District Urban	6.UG- Public District Rural	7.UG- Public District Rural	8.UG- Public District Rural	9.UG- ^a^ District Rural	10.UG- ^a^ District Rural	11. INDO- Public Tertiary Urban	12. INDO- Public Tertiary Urban	13. INDO- Public Regional Urban	14. INDO- Public District Urban	15. INDIA- ^a^ Tertiary Urban	16. INDIA- Public Tertiary Urban
Neonatal Unit Bed Strength	50	40	30	6	6	8	3	3	4	2	55	70	32	66	76	80
Total Physicians ^b^ (Certified pediatricians)	23 (7)	7 (7)	11 (3)	6 (1)	6 (2)	2 (1)	1 (0)	1 (0)	1 (0)	1 (0)	8 (8)	12 (5)	5 (5)	7 (1)	10 (7)	7 (7)
Total Nursing personnel^b^ (nurses, midwives and nursing assistants)	23	13	9	19	8	11	13	12	13	12	86	105	16	68	79	52
No. of beds per nurse, day shift	6.3	13.3	15	1	2	2.7	1	1.5	1.3	0.7	1.8	3.5	4	3.7	4	5.7
Night shift doctors	2 + 1 on call	2 + 2 on call	1 on call	1 on call	1 + 1 on call	1 on call	1 on call	1 on call	2	1 on call	1	5	1 on call	1	3 + 1 on call	6 + 1 on call
No. of beds per nurse, night shift	12.5	20	30	2	6	4	1.5	1.5	1.3	1	3.9	3.5	4	4.7	5.1	8.9

^**a**^ - Private, Not for Profit Hospital; *UG* Uganda, *Indo* Indonesia; ^b^- Physicians and nursing personnel do not include trainees

### Resuscitation at birth and safe oxygen availability

Each facility had a newborn baby corner in the delivery room where resuscitation of babies and basic essential newborn care could be provided. Skin-to skin contact soon after birth was practiced in all the facilities. Delayed clamping or milking of the cord was done regularly, except in 2 hospitals in Uganda (#1 and #2) and one in India (#15). All had self-inflating bags, 220–240 mL and masks, sizes 1 and 0. Four Ugandan hospitals (# 7–10) and the two Indian hospitals also had 500 mL self-inflating bags for term babies. In the delivery room, a common suction machine was used for both mother and baby in all the hospitals in Uganda and Indonesia; in India, suction equipment was separate for the mother and baby. While most of the hospitals confirmed the presence of low pressure (≤100 mmHg) suction machines, all the hospitals in Uganda could not provide information on suction pressure. Suction bulbs were the conventional rubber bulbs and were available in all the hospitals. Eight hospitals in Uganda had, additionally, the Laerdal ‘penguin’ suction device, which is translucent and can be opened for cleaning and disinfection/sterilization.

Oxygen was available in all the centers in the labor and delivery rooms as well as in the NU, either through oxygen cylinders and/or oxygen concentrators or piped in from a central oxygen supply source. None of the hospitals in Uganda had the capability to provide blended air and oxygen, while this was available in all the facilities in Indonesia and India. All hospitals, except for one hospital in Uganda, could provide CPAP. In Uganda, 5 facilities had 1 pulse oximeter each, all functional. All hospitals in India and Indonesia had pulse oximeters, between 18 to 50 in number. In two hospitals in Indonesia, 12 of 37 (32%) in #12 and 4 out of 18 (22%) in # 14 were not in working order.

### Temperature maintenance and kangaroo mother care (KMC) (Table 3)

All hospitals except for 4 in Uganda provided separate caps/hats in the labor and delivery rooms and in the NUs.

**Table 3:** KMC and Equipment in Neonatal Units

Type of Hospital	1.UG- ^a^ Tertiary Urban	2.UG- Public Regional Urban	3.UG-Public Regional Urban	4.UG- Public District Urban	5.UG- Public District Urban	6.UG- Public District Rural	7.UG- Public District Rural	8.UG- Public District Rural	9.UG- ^a^ District Rural	10.UG- ^a^ District Rural	11.INDO -Public Tertiary Urban	12.INDO- Public Tertiary Urban	13.INDO -Public Regional Urban	14.INDO-Public District Urban	15.INDIA- ^a^ Tertiary Urban	16.INDIA- Public Tertiary Urban
Annual Admissions	2463	1729	1707	1164	506	1586	240	300	180	156	1600	1028	970	1900	3115	2188
No. of incubators (functional)	20 (15)	2 (0)	11 (3)	3 (3)	0	2 (0)	3 (3)	1 (1)	2 (2)	2 (2)	40 (40)	64 (55)	43 (39)	56 (40)	7 (7)	8 (8)
Radiant warmers (functional)	5 (1)	2 (2)	4 (4)	1 (1)	1 (1)	0	1 (1)	0	0	0	15 (15)	13 (11)	14 (13)	6 (6)	42 (42)	72 (72)
KMC with mothers staying in the hospital	Yes	Yes	Yes	Yes	Yes	Yes	Yes	Yes	Yes	Yes	Yes	Yes	No	No	Yes	Yes
KMC during mothers’ visits	No	No	No	No	No	No	No	No	No	No	Yes	Yes	Yes	Yes	Yes	Yes
Infusion pumps	Yes	Yes	No	No	Yes	No	No	No	No	No	Yes	Yes	Yes	Yes	Yes	Yes

^**a**^ - Private, Not for Profit Hospital; *UG* Uganda, *Indo* Indonesia

KMC was practiced to some extent in all the hospitals, with mothers staying in the unit/hospital (all 10 in Uganda; 2 in Indonesia, and 2 in India). It was also practiced during the mothers’ hospital visits in all except in Uganda.

Hospital # 15 in India also practiced “family centered care”. Parents were allowed 24-h access to their babies, and mothers after discharge stayed in a room near the NU and were available to provide KMC and expressed breast milk. Once the babies were stable and/or about 1500 g, mothers were additionally involved in the care of their baby, such as baths and feeding.

NUs utilized both incubators and radiant warmers or open care systems (Table 3). Incubators were used more often than radiant warmers in Uganda, whereas in Indonesia, units had both incubators and radiant warmers. In India, there was a distinct preference for locally manufactured radiant warmers. Ninety-six per cent of radiant warmers and 82.6% of incubators in all the facilities studied were in functioning order.

Monitoring of the baby’s temperature through the axillary route was primarily through use of shared digital thermometers that were stored dry and swabbed with alcohol between babies. Separate thermometers for each baby were available only in three hospitals in Indonesia, and in both the Indian hospitals.

### Feeding and IV fluids

All the hospitals used expressed human milk for feeding the babies, but proportions of human milk and formula substitution were not available. Only one of these hospitals (in India, # 15) had a milk bank supporting the unit. Two hospitals in Uganda (#1 and # 6); all in Indonesia and one in India (#16) stored surplus expressed breast milk in refrigerators in the unit. Hospital # 15 in India had mothers near the unit as part of associated family centered care. All the facilities had resources to provide gavage feeding. Most, except for two in Indonesia (# 12 and # 13), also used cups for feeding small babies. In India, the local traditional “paladai” (a cup with a small open spout/trough) was used.

All facilities had resources to administer intravenous fluids when required. They reported use of single-use cannulas and intravenous infusion sets that were changed between 24 and 72 h. Three Ugandan hospitals (#1, #2, # 5), and all in Indonesia and India had infusion pumps. The remaining Ugandan hospitals used adult infusion sets and did not have infusion pumps, micro-burettes or flow regulators. Only three hospitals (in Indonesia, #12, #13 and # 14) had commercial premixed intravenous fluids. All other units mixed intravenous fluids, usually within the unit. In one unit in Indonesia (#11), the fluids were prepared in the pharmacy.

### Prevention of infection (Table 4)

All hospitals, except for #8 in Uganda, reported having 24 h running water. All had liquid soap and sinks for hand washing with 13 having a sink in every room with babies. All except 5 in Uganda (# 4, #7–10) had alcohol-based hand-rubs; five (# 1–3, #5,6) had limited supplies, placing them only at selected sites, such as near incubators.

**Table 4:** Infection Prevention

Type of Hospital	1.UG- ^a^ Tertiary Urban	2.UG- Public Regional Urban	3.UG- Public Regional Urban	4.UG- Public District Urban	5.UG- Public District Urban	6.UG- Public District Rural	7.UG- Public District Rural	8.UG- Public District Rural	9.UG- ^a^District Rural	10.UG -^a^District Rural	11.INDO-Public Tertiary Urban	12.INDO- Public Tertiary Urban	13.INDO- Public Regional Urban	14.INDO- Public District Urban	15.INDIA-^a^ Tertiary Urban	16.INDIA- Public Tertiary Urban
Alcohol hand rub	Select places	Select places	Select places	No	Select places	Select places	No	No	No	No	Yes	Yes	Yes	Yes	Yes	Yes
Linen Source	Hospital	Family	Family	Family	Family	Family	Family	Family	Family	Family	Hospital	Hospital	Hospital	Hospital	Hospital	Hospital
Handling of linen	HW, LD	HW, LD	MW, MD	HW; LD/ DG	HW, LD	HW, LD	HW; LD/ DG	HW; LD/ DG	HW; LD/ DG	HW; LD/ DG	HW, LD	MW, MD	MW, MD	MW, MD, A	MW, MD	MW, MD,A
Reproces- sing of suction bulb	Washed ^b^	Washed ^b^	Washed ^b^ and wiped with alcohol	Washed^b^	Washed ^b^	Soaked in “jik”^c^	Washed^b^	Washed^b^	Washed^b^	Washed^b^	Washed^b^	X	X	Washed^b^	Single use	X
Reproces-sing of bag and mask	Only mask washed^b^	Only mask washed ^b^	Bag and mask wiped with “jik”^c^	Only mask dipped in “jik” ^c^	Only mask washed^b^	Only mask washed ^b^	Only mask dipped in “jik” ^c^	Only mask dipped in “jik” ^c^	Only mask dipped in “jik” ^c^	Only mask dipped in “jik” ^c^	Only mask washed ^b^	Disassembled and sterilized every time	Disassembled and sterilized every time	Only mask cleaned with alcohol	Only mask washed^b^	Disassembled and sterilized once a day
Use of antibiotics	1 vial for each baby	shared vial	shared vial	shared vial	shared vial	1 vial for each baby	shared vial	shared vial	shared vial	shared vial	shared vial	1 vial for each baby	shared vial	shared vial	shared vial	shared vial

X – Not applicable; ^**a**^ - Private, Not for Profit Hospital; *UG* Uganda, *Indo* Indonesia, *HW, LD* Hand-washed, line dried, *HW; LD/DG* Hand-washed, line-dried/ dried on ground, *MW,MD* Machine washed, machine dried, *MW,MD,A* Machine washed, machine dried and autoclaved ^b^- Washed with soap and water; ^c^ - Jik is a chlorine-based solution into which the item is dipped or soaked for decontamination; after which it is washed with soap and water

Routine cleaning of the floor with mopping was carried out daily in all the centers. All the hospitals had dedicated containers for ‘sharps’, waste receptacles with lids and plastic linings and incinerators.

Baby linen was supplied by the facility in 6 hospitals, while the 10 hospitals in Uganda requested mothers/families to bring the linen. In the 10 hospitals in Uganda, linen was washed by hand and dried on lines or on the ground in open air; in the remaining 6 (in Indonesia and India), it was machine washed and dried. In one hospital in Indonesia (#14) and one in India (#16), the linen was additionally autoclaved centrally.

Among the 15 facilities that used rubber suction bulbs (all in Uganda and Indonesia and 1, #15, in India), 8 in Uganda and 2 in Indonesia reused suction bulbs after merely washing them with soap and water. One hospital in Uganda (#6) dipped it in a chlorine based solution “jik”, and washed it with soap and water. Only one hospital in India discarded them after every use. With the exception of 2 hospitals in Indonesia (#12 and 13), none of the facilities reprocessed the self-inflating bag and mask with full disassembly and reassembly after each use. One hospital in India reprocessed it in this manner once a day. In 13 facilities (all 10 in Uganda, #11 and #14, in Indonesia and #15 in India), health workers simply detached the mask and cleaned it with soap and water, a chlorine- based solution or alcohol.

### Treatment of infection

Regarding antibiotics, Ampicillin/ Penicillin and Gentamicin were available in all NUs (Additional file 2: Table S2). In addition, hospitals had access to cephalosporins, vancomycin and carbapenems. A larger variety of antibiotics were available in the facilities in India and Indonesia. Antibiotics were generally available in multi-dose vials. In 2 facilities in Uganda (# 1 and # 6), one vial was reserved for one baby. In the others, they were shared between babies. One mL syringes, that are usually recommended for accurate dosing of small volumes in babies, were not available in 7 hospitals in Uganda.

#### Management of Jaundice and Blood Transfusions

Phototherapy units were available in all the hospitals. Most hospitals had resources for blood transfusion, except for two facilities in Uganda, and all except 8 hospitals in Uganda could carry out exchange transfusions.

#### Laboratory tests

All hospitals had resources to carry out blood group typing, blood glucose, and blood counts, and all but 7 facilities in Uganda (#4–10) could perform serum bilirubin levels, serum electrolytes and blood cultures.

## Discussion

This study highlights the variability of facility readiness for care of “small and sick babies” in referral hospitals in LMIC. Although the units were all locally referred to as “Neonatal Intensive Care Units” (NICU), they actually consisted of a mixture of special care (Level 2 or SCNU) and Intensive care (Level 3 - NICU) units. Even when viewed through the lens of the requirements of SCNU, many centers including “tertiary” centers had challenges.

Interestingly, we noted that in the NUs in the hospitals that had records of birth weights, 51.7% of the babies admitted weighed 2500 g or more. In the authors’ experience, not all the normal weight term infants admitted to the NU are sick. Some just require extra observation and monitoring; for example, respiratory rates or blood sugar estimations, in the initial stages after which they are transferred out to the mothers in the post-natal wards. Some late preterm babies too may weigh more than 2500 g. We believe the term “high risk/small and sick babies” may be more appropriate than the conventional “small and sick babies”, when dealing with newborns needing extra care, in LMIC. It is possible that the greater numbers of admissions were partly due to the high-risk nature of these pregnancies and because of limited monitoring capabilities for babies in the postnatal ward.

In 5 of 8 facilities with the data, more than a third of inborn babies were admitted to the neonatal units (Table 1). As noted earlier, some of these admissions were to provide “extra rest of the mothers”. While this may be important, it adds to the workload of the already over-burdened staff in the NUs, results in overcrowding, needless use of formula feeds and potential risk of cross- infections. 

### Bed strength and human resources

Professional bodies, such as the American Academy of Pediatrics), the National Neonatology Forum of India), organizations such as UNICEF, India and Ministries of Health in some LMIC, such as the Ministry of Health and Family Welfare, India have developed recommendations on bed strength, spacing and staffing [8, 10–12]. Recommendations are often based on the number of deliveries and do not in general, consider out-born admissions, that add to the workload, both by numbers and also with acuity. As shown in Table 2, hospitals in Indonesia and India had higher bed strengths, but the numbers of neonatal beds were much lower in most of the Ugandan hospitals. Placing multiple babies in a cot is not uncommon in LMIC. This carries increased risk of infection and places an additional strain on the care providers.

In this study there was a higher proportion of Pediatricians and nurses in Indonesia and India than in Uganda, but even in these facilities, the nurse -patient ratio was inadequate. Shortage of nursing staff was a challenge with one nurse covering 15 to 30 babies in 3 of the hospitals in the night shift. In LMIC, due to inadequate skilled staff, task-shifting to or task-sharing with trained but less skilled staff or even family members for some of the non-specialized care may be essential until the countries can afford to provide adequate staff [13].

With increasing proportion of facility births and high mortality among preterm and low birth weight babies, there is growing need for focus on the special requirements of high-risk/small babies and those with problems. Thus, while the families, community health workers and midwives play important roles, there is an increasing need for Pediatricians/Neonatologists and Pediatric/neonatal nurses with special expertise and interest in newborn care. Shortage and maldistribution of suitable health care providers have also been highlighted as key issues in other studies on newborn care from India and Indonesia [14, 15].

### Transport

We noted that although many hospitals had ambulances, they were not used for transporting babies from the birth place at home or the peripheral centers to the referral hospitals. When babies are brought into the referral centers, they are mostly accompanied by the mother and other family members, but have not had the benefit of stabilization before or during transport. Although skin to skin contact may provide some temperature support, without additional care during transport, some of these vulnerable babies may reach referral centers in moribund states. Mapping of suitable referral centers, appropriate dissemination of this information and use of mobile phones may assist in initial stabilization of babies with problems and better facilitate transport to the appropriate referral center. Ideally, the at- risk mothers and those with anticipated deliveries of small babies should be referred in a timely manner to suitable referral centers.

### Maintenance of records and effective use and reporting of data

Data maintenance was a major challenge. For instance, in the Ugandan hospitals, birth weight was not available for all babies and gestational age was often not noted. While some of the information may be on individual case sheets, they were not consistently or correctly available in registers or in the consolidated facility records that fed into the country health information systems (HIS). Since “complications of prematurity” is widely recorded as the most common cause of neonatal mortality, this deficiency has serious implications. Accurate data is critical for the HIS, for internal review (such as perinatal/neonatal death audits), as well as for improving quality of care.

Neonatal mortality showed significant variations in the different facilities. Some of the smaller facilities with fewer admissions had a lower mortality probably due to fewer complicated cases and some sick babies being sent to the larger centers. Comparison of mortality rates across hospitals is difficult because of a variety of influencing factors. While quality of care is important, it is well known that the types of babies cared for also matter. For example, out-born admissions are often sicker and have higher mortality rates [16]. A more comprehensive assessment of neonatal mortality can be obtained by evaluating stillbirths, particularly, fresh/intrapartum stillbirths, since in some centers, certain neonatal deaths at birth may be recorded as stillbirths [17]. In some centers in Uganda, fresh stillbirth rates were reported to be as high as 65.5% of total stillbirths.

Mortality rates also may be under-reported where babies are discharged against medical advice (DAMA). DAMA accounted for 0.4 to 20% of admissions. The outcome of these babies is unknown but may be presumed to be poor, since a major factor in DAMA is the fact that the baby is so sick that the relatives feel that further care is futile. Other potential reasons for DAMA include non-affordability of care where payment is required, inadequate communication strategies and poor infrastructural support to relatives [18].

Additionally, data on neonatal deaths may be under-reported when out-born babies are admitted in the Pediatric wards and not in the NU. These are frequently babies who have suspected or frank infections and have a higher risk of dying. These deaths may not be included in the total facility newborn deaths, unless such deaths in the Pediatric wards get disaggregated according to age and these newborn deaths get consolidated with the deaths from the NU, a process that may be challenging.

### Components for basic neonatal resuscitation

A newborn corner in the delivery room, and reusable self-inflating bags with masks were present in all the hospitals studied. Some facilities used a common suction machine for mothers and babies in the delivery room, which carries the potential risk of excessive negative pressure, leading to apnea and bradycardia in the baby if pressure is not adjusted appropriately with each use. Similarly, having larger, 500 mL. bags carry the risk of volume trauma, as even the normal weight term baby requires only 6–8 ml/kg of tidal volume for which the conventional newborn size bag (220–240 ml) is more than adequate [19]. Care providers need to be trained and supervised/mentored to achieve adequate mask seal and provide appropriate ventilation [20].

### Safe use of oxygen

Many facilities in LMIC, as in the Ugandan hospitals in this study, depend on oxygen from cylinders or oxygen concentrators without blenders. The capability to provide appropriate concentrations of oxygen and consistent monitoring with pulse oximeters, are essential to reduce retinopathy of prematurity (ROP). India contributes to 10% of worldwide cases of blindness and visual impairment from ROP, highlighting the importance of safe oxygen use; prevention being far better than cure [21].

### CPAP

CPAP was available in almost all the centers. CPAP also requires the safe administration of oxygen, as only select equipment, such as the Pumani bubble CPAP, have the capability of in built regulation of oxygen concentration (Website-http://hadleighhealthtechnologies.com/pumani-bcpap/).

### Kangaroo mother care (KMC)

The immediate and long-term advantages of KMC have been well documented [22]. All facilities practiced KMC to some extent, but implementation needs to be further strengthened and expanded. The value of mothers or female relations in the care of these babies has been documented earlier in LMIC as in India with beneficial effects and without necessarily causing increased infection. It can also help empower women resulting in earlier discharge and, subsequently, better care at home [23, 24] More recently, Family Centered Newborn Care (FCNC), a “client focused “practice to promote and support a partnership between the health care team and the family, with mothers/family members providing selected non-specialized care for babies is gaining increasing acceptance around the world. In a randomized controlled trial, the intervention reduced health care providers’ workload and increased family members’ competence in infant care at home. There was no difference in nosocomial infections between the groups, and it also promoted pre-discharge exclusive breastfeeding [25].

### Extra support for temperature maintenance

Other methods of temperature support in this study included overhead radiant warmers or open care systems and incubators [26]. Overhead warmers, available in a number of varieties including more economical versions, allow ready access to the baby and, in general, are easier to clean and maintain. They are associated with increased insensible water loss, but this can be overcome to some extent with the administration of additional fluids, especially in the weight and gestational age groups that often constitute the priority in LMIC. Studies on associated morbidities including infections have been variable but the numbers evaluated have been small and have not shown any significant differences [27]. Temperature monitoring is important and axillary temperature is more commonly used, as was noted in this study, with less risk of trauma and cross infection [28]. Despite the fact that thermometers are low cost commodities, only 5 facilities used a separate thermometer for every baby.

### Procurement and maintenance of essential commodities

All the facilities studied had equipment and supplies required for basic essential newborn care, including in the delivery room, and met some of the requirements for high risk and sick babies. Every Woman, Every Child (2012) through the UN Commission for Life Saving Commodities, identified four products for newborn care, namely, antenatal corticosteroids for preterm births, chlorhexidine for cord care, equipment for basic resuscitation and antibiotics for treatment of neonatal sepsis [29]. While this brought attention to therapies that were not universally available or utilized appropriately, it also tended in some cases to inadvertently promote a ‘magic bullet’ approach instead of more comprehensive components covered in this study [30]. Although this study showed that most of the equipment was functional, maintenance of equipment, particularly donated equipment, is often a challenge in many facilities in LMIC. Inadequacy of equipment and commodities has also been reported to be a significant problem in a number of facilities in India and Indonesia [14, 31].

### Feeding of high risk and small babies

It was encouraging that in all facilities mothers provided breast milk. Expressed human milk especially from their own mothers, has considerable advantages for preterm and low birth weight babies [32]. Milk banks are costly to establish and operate, require stringent quality control, and are available to a limited extent in LMIC. In this study, only one hospital in India had a milk bank. However, all the hospitals had the capability to store extra expressed breast milk for short periods in the refrigerator.

Small and sick babies tolerating feeds may be unable to suck adequately and need additional support in the form of tube feeding and, subsequently through use of cups. In India, the “paladai”, a traditional cup with an open spout or a “trough” like extension, is extensively used for feeding small babies and has distinct advantages over the conventional cup, with less spillage, and more ready acceptance by babies [33]. Challenges are also particularly high for achieving exclusive breastfeeding for babies who are separated from their mothers. Lactation consultants are not widely available in LMIC. It may be necessary to task-shift and train alternative workers to provide some basic lactation support for mothers in SCNU along with the nurses and physicians. Avoidance of bottles, not only for feeding but also for collection of human milk is beneficial as its presence sends a wrong message.

### Management of jaundice

Neonatal jaundice is a major cause of morbidity in LMIC. Early detection at the community and facility levels, effective phototherapy with appropriate wavelength of light with monitoring of serum bilirubin levels can decrease the need for exchange transfusions. Innovative methods for early detection and management of jaundice are also essential [34, 35]. In countries with advanced health care systems, exchange transfusions have become uncommon due to earlier detection of jaundice and effective phototherapy. In contrast, exchange transfusions are far more common in LMIC. Hence, it is worth looking into ensuring that selected well-functioning SCNUs even at district levels have the resources and capabilities to manage jaundice effectively and perform exchange transfusions.

### Prevention of infection

All hospitals in this study except one, reported having 24 h running water supply and resources for waste disposal including incinerators. Some of the essential elements of infection prevention include proper cleaning of surfaces, adequate supplies of clean linen, single use items, use of breast milk, safe administration of injections and intravenous fluids, and capability for identification of causative microbes along with susceptibility testing; areas in which there may be significant deficiencies in supplies and practices [36].

In most of the hospitals in Uganda, the family was expected to bring linen for their babies. The effectiveness of laundering procedures used in cleaning the linen was unclear. Hand washing linen and drying on lines or on the ground carry greater risks in contrast to machine washing and drying. It is essential that facilities supply clean linen kept ready for use.

We found that reprocessing resuscitation equipment was inadequate and inconsistent in the centers studied. Most used rubber suction bulbs, which cannot be opened to permit proper cleaning, and generally reused them after cleaning with just soap and water. Eight hospitals in Uganda had the translucent bulbs that could be opened. Except for two hospitals in Indonesia, there was minimal compliance with recommended reprocessing of resuscitation devices with disassembly, initial decontamination, proper cleaning with soap and water and high-level disinfection such as boiling or sterilization by autoclaving, followed by reassembly and safe storage [37]. These recommendations, however, can present significant challenges in being time-consuming and in having potential risks of losing small parts, resulting in poor compliance.

There may be a significant underestimation of infection relevant to newborn health in LMIC. The term “complications of prematurity” as a cause of death is likely to include infections in addition to conditions such to respiratory distress syndrome, apnea and intraventricular hemorrhage. Thus, the proportion of babies dying of infections is probably greater than what is generally portrayed, especially as diagnosis of infections poses challenges. A high incidence of neonatal sepsis has been reported in some hospitals [38]. When quality of care is poor, preterm babies may die more often due to initial problems such as birth asphyxia or breathing difficulties. However, as care improves and babies survive for longer periods, prevention of nosocomial infection assumes an even greater importance.

### Treatment of infection

The facilities studied had access to a number of essential antibiotics, but it was not within the scope of this study to determine actual antibiotic usage. Antibiotic resistance has grown to the extent that options for appropriate alternatives are limited and may not always be available. Countries should establish national guidelines for antibiotic therapy based on their local distribution of organisms and antibiotic sensitivity patterns, with periodic review as required. Appropriate antibiotic stewardship with avoidance of needless administration is also essential to decrease antimicrobial resistance.

### Intravenous fluid administration

Although all facilities had resources for administration of intravenous fluid therapy, most had to mix their fluids, with potential risk for infection. Additionally, most facilities in Uganda had only adult infusion sets, through which it is difficult to safely infuse the small volumes required by babies.

### Limitations

This study provides a snapshot of some of the resources in a limited number of referral facilities caring for high risk/ small and sick newborns. Variations may be even greater in facilities in LMIC. The data were self-reported, and basic components such as weight of babies and gestational age were not available for some centers. These are not only challenges but highlight the existing status in some centers, notably in Sub-Saharan Africa.

### Additional elements to be considered

While the physical requirements related to at-risk, small and sick baby care are important, in this study we were unable to evaluate certain additional components noted below that can have further impact on neonatal mortality.

### Improving quality of care (QoC)

WHO with partners has highlighted the importance of compassionate and good QoC at facility level [39]. In many LMIC, there is often a significant variation in the existing quality of care, with some hospitals providing excellent care but with many others having neither the required skills, supplies nor the motivation, resulting in poorer quality care including inadequate pain management [40].

### Health system strengthening with links between facilities and communities

Facility care is also incomplete without health system strengthening. In addition, facility/ community links, supportive supervision of trained community health workers and participatory involvement of the community through multiple strategies is essential.

### Prioritizing low hanging fruits

As funds will not always meet the demand, it is essential that decisions are taken involving key stake holders to prioritize the order and the extent to which the various components need to be implemented, keeping equity and costing in mind.

### Too little, too late; too much, too soon

As has been noted in maternal care, there is always a risk of doing too little too late and too much too soon [41]. An example of the former is, despite promoting facility deliveries to improve outcome, emphasis in some areas has still focused only on “essential newborn care” and the preparation of facilities to go further to care for the at-risk and sick babies has been inadequate. At the same time, progressing too quickly to instituting intensive care including mechanical ventilation and related interventions, without having the basic resources and skills to ensure competent, compassionate, quality based special/level II care, with adequate infection prevention, may have undesired effects. In establishing appropriate care for the newborn in whom, among other issues, the brain is still developing, the well-known dictum “Do no harm” has a very special significance.

## Conclusion

We show that referral NUs in LMIC have significant challenges in meeting some of the basic requirements of Level II / special care neonatal units. The high neonatal mortality was responsible for some countries not achieving Millennium Development Goals 4. It is likely that newborn health may still continue to be one of the most challenging components and even be an important deterrent to the achievement of Sustainable Development Goals 3.2.2, unless a concerted effort is made to provide comprehensive, high quality care for the newborn both at the facility and community levels.

## Additional files

Additional file 1: Facility readiness data collection tool (level II newborn care). (XLSX 29 kb)

Additional file 2: **Table S2.** Antibiotic Availability. (DOCX 17 kb)

## Abbreviations

CPAP: Continuous positive airway pressure
DAMA: Discharged against medical advice
FCNC: Family centered newborn care
HIS: Health information system
KMC: Kangaroo mother care
LMIC: Low and middle-income country/countries
NICU: Neonatal intensive care unit(s)
NU: Neonatal unit(s)
SB: Stillbirth(s)
SCNU: Special care newborn unit(s)

## Authors’ information

IN and JR have experience in neonatal intensive care units in high and low and middle-income countries; IN has worked in addition in the area of global health with special relevance to maternal and newborn health and nutrition for over 20 years. JNS, being the Asst. Commissioner, Child Health, Ministry of Health has experience in major policy decisions related to in-country newborn and child care; and SL, RR, NT, CNK, VNK, PS have experience in facility-based care of the high-risk/small and sick newborns in LMIC and HYC experience in a high-income country and collected data.. PW, has been involved in facility based newborn care in Uganda and has special experience in assessing facility readiness. JJW, SD, DK, PM and EO collected and consolidated data.
