# Supplementary material for: Facility readiness in low and middle-income countries to address care of high risk/ small and sick newborns
Source: Matern Health Neonatol Perinatol. 2019 Jun 18;5:10. doi: 10.1186/s40748-019-0105-9 (PMC6580648; doi:10.1186/s40748-019-0105-9)
Supplement: Supplementary file 2 — Table S2. Antibiotic Availability. (DOCX 17 kb) [file 40748_2019_105_MOESM2_ESM.docx]

| **Additional file 2: Table S2. Antibiotic Availability** | | | | | | | | | | | | | | | | |
| --- | --- | --- | --- | --- | --- | --- | --- | --- | --- | --- | --- | --- | --- | --- | --- | --- |
| **Type of Hospital** | **1.UG- § Tertiary Urban** | **2.UG- Public Regional**  **Urban** | **3.UG- Public Regional**  **Urban** | **4.UG- Public District**  **Urban** | **5.UG- Public District**  **Urban** | **6.UG- Public District Rural** | **7.UG- Public District Rural** | **8.UG- Public District Rural** | **9.UG- §**  **District Rural** | **10.UG- §**  **District Rural** | **11.INDO Public Tertiary Urban** | **12.INDO Public Tertiary Urban** | **13.INDO**  **Public Regional Urban** | **14.INDOPublic District Urban** | **15.INDIA§**  **Tertiary Urban** | **16.INDIAPublic Tertiary Urban** |
| **Ampicillin** | √ | √ | √ | √ | √ | √ | √ | √ | √ | √ | √ | √ | √ | √ | √ | √ |
| **Penicillin** |  | √ | √ | √ | √ |  | √ | √ | √ | √ | √ | √ | √ | √ | √ | √ |
| **Ampicillin- sulbactam** |  |  |  |  |  |  |  |  |  |  | √ | √ | √ | √ | √ | √ |
| **Cloxacillin** |  | √ |  |  |  |  | √ | √ | √ | √ | √ | √ | √ | √ | √ | √ |
| **Gentamicin** | √ | √ | √ | √ | √ | √ | √ | √ | √ | √ | √ | √ | √ | √ | √ | √ |
| **Amikacin** | √ |  |  |  |  |  |  |  |  |  | √ | √ | √ | √ | √ | √ |
| **Cotrimoxazole** |  | √ | √ |  | √ | √ |  |  |  |  | √ | √ | √ | √ | √ | √ |
| **Azithromycin** |  | √ |  |  | √ |  |  |  |  |  | √ | √ | √ | √ | √ | √ |
| **Vancomycin** |  |  |  |  | √ |  |  |  |  |  | √ | √ | √ | √ | √ | √ |
| **Cefotaxime** | √ |  | √ |  | √ |  |  |  |  |  | √ | √ | √ | √ | √ | √ |
| **Cefuroxime** |  |  |  |  | √ |  | √ | √ | √ | √ | √ | √ |  | √ | √ | √ |
| **Cefixime** |  |  | √ |  | √ | √ |  |  |  |  | √ | √ |  | √ | √ | √ |
| **Ceftazidime** |  |  |  |  |  |  |  |  |  |  | √ | √ | √ | √ | √ | √ |
| **Ceftriaxone** | √ | √ | √ | √ | √ | √ |  |  |  |  | √ | √ | √ | √ | √ | √ |
| **Nalidixic acid** |  | √ | √ |  | √ | √ |  |  |  |  | √ | √ |  | √ | √ | √ |
| **Ciprofloxacin** |  |  | √ |  | √ | √ |  |  |  |  | √ | √ |  | √ | √ | √ |
| **Carbopenem** |  |  | √ |  |  |  |  |  |  |  | √ | √ |  | √ | √ | √ |
| **Meropenem** |  |  |  |  | √ |  |  |  |  |  | √ | √ | √ | √ | √ | √ |
| **Colistin** |  |  |  |  |  |  |  |  |  |  | √ | √ |  | √ | √ | √ |
| **Pipercillin- tazobactam** |  |  | √ |  | √ |  |  |  |  |  | √ | √ |  | √ | √ | √ |
| **Targocid** |  |  |  |  |  | √ |  |  |  |  | √ | √ |  | √ | √ | √ |
| **Linezolid** |  |  |  |  |  |  |  |  |  |  | √ | √ |  | √ | √ | √ |
| √ - Available; § - Private, Not for Profit Hospital; UG – Uganda; Indo – Indonesia. | | | | | | | | | | | | | | | | |
